# The effect of 8 weeks aerobic exercise on severity of physical symptoms of premenstrual syndrome: a clinical trial study

**DOI:** 10.1186/s12905-018-0565-5

**Published:** 2018-05-31

**Authors:** Zahra Mohebbi Dehnavi, Farzaneh Jafarnejad, Somayeh Sadeghi Goghary

**Affiliations:** 1 Department of Midwifery and Reproductive Health, Faculty of Nursing and Midwifery, Esfahan, University of Medical Sciences, Esfahan, Iran; 2 Faculty of Nursing and Midwifery, Mashhad, Iran; 3 Faculty of Nursing and Midwifery, Mashhad University of Medical Science, Mashhad, Iran

**Keywords:** Premenstrual syndrome, Aerobic exercise, Physical symptoms

## Abstract

**Background:**

Premenstrual syndrome (PMS) is a common disorder among women of reproductive age. Nearly 40% of women report problems with their menstrual cycles. Exercise is one of the recommended treatments to reduce symptoms of premenstrual syndrome (PMS). The present study was conducted to determine the effect of 8 weeks aerobic exercise on severity of physical symptoms of premenstrual syndrome (PMS).

**Methods:**

This study was a randomized clinical trial (IRCT2015021721116N1) that was performed on 65 students living in student dormitories of Mashhad University of Medical Sciences in 2016, Iran. Samples were randomly assigned to control and intervention groups. The intervention group engaged in 8 weeks of aerobic exercises, three times a week, and 20 min for each session. The tools were research unit selection questionnaire, midwifery and personal particulars, temporary determination of premenstrual syndrome, Beck Depression, recorded daily symptoms of premenstrual syndrome and Borg scale. We analyzed the data using SPSS software and Mann–Whitney U test and Friedman test.

**Results:**

At the beginning of the study, both control and intervention groups were homogeneous. The results of independent t-test showed that among the physical symptoms of the premenstrual syndrome in the intervention group compared to the control group, at the end of the study, headache (*p* = 0.001), nausea, constipation diarrhea (*p* = 0.01), swollen (*p* = 0/001) had a significant reduction. Also, the comparison of the difference between the mean of the signs at the beginning and the end of the study, bloating (*p* = 0.01), Vomiting (*p* = 0.002), hot flashes (*p* = 0.04), increase in appetite (*p* = 0.008) were significantly decreased.

**Conclusion:**

Aerobic exercise as one of the ways to treat premenstrual syndrome can reduce the physical symptoms of the syndrome.

**Trial registration:**

Name of registry: Zahra Mohebbi Dehnavi.

IRCT registration number: IRCT 2015021721116 N1.

Registration date: 2015 − 08-28.

Registration timing: retrospective.

## Background

Menstrual cycle is one of the most important signs of reproductive system functioning in females, but sometimes this phenomenon is associated with signs and symptoms that cause physical and psychological problems for women. PMS refers to a set of repetitive symptoms that begins at the end of the secretion phase of the menstrual cycle (5–7 days before menstruation) and ends in the follicular phase (2–4 days after menstruation) [1]. For this syndrome, more than 150 signs have been identified, some of which are: abdominal cramps, boredom, abdominal bloating, pain and tenderness of the breasts, acne, back pain and headache, joint pain and muscle pain, weight gain, energy shortages, Changes in appetite and thirst, constipation, heart rate elevation [2, 3]. Due to the high prevalence of this syndrome and the role of women in the family and community, treatment is important. The prevalence of these symptoms varies according to factors such as culture, attitude, age, exercise, nutrition, and underlying diseases [1].

Given that today, a large number of women are involved in the fields of occupation, education, family, and other responsibilities. And the stress associated with these responsibilities is related to health issues, such as PMS, lifestyle changes, membership in supportive groups, the use of stress control methods along with supplementary drugs and vitamins may help relieve the symptoms of the disease But information on the complete treatment of this disease is currently very limited [4]. Several possible causes have been reported for the syndrome, including: estrogen, progesterone, fluid retention, hyperprolactinemia, vitamin B6 deficiency, hypoglycemia, prostaglandin deficiency, androgen hormones allergy, psychosomatic problems, increased activity of aldosterone and renin plasma, thyroid disorders and lack of serotonin [5–7]. Since precise pathophysiology of PMS is not known, there is no definitive treatment for it, and most of the symptom is being treated. Generally, treatments can be arranged in three groups: Surgical treatment, drug treatment (such as hormonal use, antidepressant and analgesic drugs) and non-drug therapies (such as lifestyle changes and exercise) [8].

Considering the side effects of drug and surgical treatments, non-drug treatments, especially physical activity, have attracted the attention of specialists and women with the disease [9]. Physical activity is a suitable method for the treatment of PMS and is the best method for all women to reduce pressure and balance in the brain’s chemical secretions. It seems that physical activity by increasing endorphins and reducing adrenal cortisol leads to improvement of symptoms of PMS, increased pain tolerance, decreased anxiety, depression and other problems [10].

Vishnupriya et al. Examined the effect of aerobic exercise with different severity (low, moderate, severe) on 61 women with PMS for 6 weeks, The results showed that symptoms of PMS were significantly decreased in moderate intensity aerobic exercise [11], But Silva and Sadler’s research reported that aerobic exercise does not affect on symptoms of PMS [12, 13], While David et al. Reported that aerobic exercise had a negative effect on symptoms of PMS [14].

### Study objectives and aims

The general objective of this study was to determine the effect of 8 weeks aerobic exercise Exercise on the severity of physical symptoms of PMS. Specifically, the study aimed to:

- The mean of indicators of physical symptoms of PMS in the control and intervention groups
- Comparison of the mean of physical symptoms of PMS in the control and intervention groups

## Methods

### Study design and setting

The present study is a randomized clinical trial with IRCT 2015021721116 N1 registration code. This study was performed on 65 female students that living in dormitories of Mashhad University of Medical Sciences in 2016.

Initially, Sample size According to El-Lithy’s study [15], 30 people in each group were estimated, with Considering sample loss, 35 people were estimated in each group. At first, randomly and according to the table, two hostels were randomly selected and in order to avoid any interference in the sampling, a dormitory was considered as the control group and one was considered as the control group. Then, in dormitories of the Mashhad University of Medical Sciences, people with symptoms of PMS were requested to attend a meeting in the dormitory’s residence According to the date announced. Then, the research objectives were explained to the participants and they were asked to complete written consent if they wished to participate in the study. The inclusion criteria included: regular menstrual cycles (cycles of 21-35 days with a bleeding time of 3–10 days), PMS (according to the interim screening questionnaire, having 4 of 11 The question mark) and Score below 40 from Beck Depression questionnaire was entered into the study. The exclusion criteria at the beginning of the study included pregnancy, participation in other sports programs, continuous use of medication, chronic disease, Women with neurological, psychological disorders, women under hormonal treatment, women with endocrinological diseases, women with local lesions causing pain as PID, severe depression (according to the depression questionnaire having a score above 40), the incidence of adverse events in the last 3 months. All research units were assured that each stage of the study could be excluded from the study and referring to the specialist if there were any problems that prevented them from studying. Exclusion criteria during the study included: dissatisfaction with the continuation of the research, pregnancy during the study, Exit the menstrual cycle from the normal order during the study, the failure to complete the questionnaire (3 consecutive days and 5 days interrupted) and the adverse and stressful during the study. In the next step, the questionnaires were completed by the research units.

The instruments used included: The questionnaires of temporary determination of PMS (set temporary PMS) (the validity of this questionnaire was confirmed in 2013 by Shakeri et al.) [2] We measured the reliability of this questionnaire according to the re-test method and the Spearman–Brown correlation coefficient as 0.79). Beck Depression questionnaires (This questionnaire with regard to the study by Jafarnejad et al. is a valid and reliable tool. A study by Jafarnejad et al. has mentioned that Beck et al. in 1996 obtained the credibility coefficient of the test, re-test, in a weekly interval as 0.93. A study by Ahmadi Tahoor in 2009 confirmed its content validity [2]. Recorded daily symptoms of PMS-(The validity of this questionnaire was confirmed in 2013 by jafarnejad et al.) [2]. The reliability of this questionnaire was by Cronbach’s alpha Internal consistency, and the reliability coefficient was calculated as 0.77). Borg scale-(It has the sufficient validity. Moreover, the validity of this questionnaire was confirmed by Azhary et al. in 2004. The reliability of the questionnaire was confirmed) [7].

We conducted this study in two stages, a stage prior to intervention (2 months) and the next stage after the intervention (2 months).

The intervention consisted of aerobic exercise training, which was taught to intervention units at a meeting face to face. At the end of the session, CDs and educational posters containing all the exercises were provided to the units of the intervention group and they were asked to perform aerobic training for 8 weeks, 3 times a week and 30 min each time. Each exercise cession included warming up movements for the first 5 min (including head movements, stretching and rotation of the shoulders and maintaining balance), cooling down for the last 5 min (movements in sitting and lying-down position to return to the initial state) and in the time between the two, aerobic exercises (kinetic movements including rotating and stretching the arms, rotating the upper body, standing-in-place movements).

The research units, at the end of each exercise session, had to check their pulse rate and record it in the Borg questionnaire. Also, they were asked to record the intensity of the exercises according to the Borg scale. Moreover, during the 8-week period, the recorded daily symptoms questionnaire was routinely completed by research units. Twice a week, the researcher made phone calls to the research units and encouraged them to do the exercises and complete the questionnaires. Once every 2 weeks, the researcher visited the volunteers to check the situation of exercise activities in the intervention group by recording their pulse rates after exercise and the Borg scale performance, i.e. after exercise the heart rate should be in the range of 120–150 and the Borg scale in the range of 12–15.

The control group during the same 8-week period recorded their daily activities without having any exercise programs and were encouraged to complete the questionnaire on a regular basis twice per week through phone calls made by the researcher. The information was provided to the participants.

### Community engagement and ethical considerations

Initially written consent was obtained from all participants in the research. Participants could withdraw at any stage of the research. This study did not endanger the participants’ health. During the research, the participants were referred to relevant specialists in the presence of severe PMS and severe depression according to the questionnaires. The information obtained from the participants was confidential. The information obtained from the study was presented to the participants at the end of the study. This research was approved by the Vice-Chancellor of Ethics Committee of Mashhad University of Medical Sciences (with the code 931232). The clinical trial code is IRCT 2015021721116 N1.

### Data management and statistical analyses

Information was encrypted and entered into SPSS (Version 23) software (IBM, SPSS Inc., Chicago, Illinois, USA). Then, descriptive statistics Independent t-test tests were used to analyze the data. *P* < 0.05 was considered statistically significant.

## Results

At the beginning of the study, 70 individuals entered the study and 65 followed through to the end of the study and 5 individuals were excluded from the study.

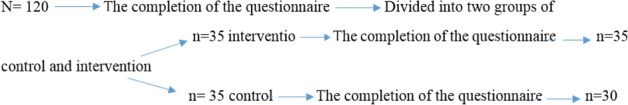



The two groups were homogeneous for demographic characteristics (Tables 1 and 2). The mean age of participants in the study in the control group was **24/06 ± 4/71** and in the intervention group was **25/22 ± 4/41**. Menstrual intervals in the control group were **7/33 ± 1/34** and in the intervention group were **6/6 ± 1/41**.

**Table 1:** Average and homogeneity of quantitative variables of participants’ demographics

Variable	Control $\overline{X}\pm SD$	Intervention $\overline{X}\pm SD$	*P*-Value^*^
Age	24/06 ± 4/71	25/22 ± 4/41	0/2
Monthly bleeding intervals	27/63 ± 3/39	28/34 ± 3/69	0/42
duration of menstrual bleeding	7/33 ± 1/34	6/6 ± 1/41	0/37
intensity of Dysmenorrhea	3/80 ± 2/85	4/02 ± 3/52	0/77

^*^P < 0.05 was considered statistically significant

^**^significant

**Table 2:** Qualitative variables of participants’ demographics

Variable	Control Number (percent)	Intervention Number (percent)	*P*-Value^*^
History of absence from the workplace or university	9 (30%)	13 (37/1%)	0/54
History of the use of treatment before the study	10 (33/3%)	16 (45/7%)	0/31
The Impact of Pre-Study Therapeutic Therapy	8 (26/7%)	11 (31/4%)	0/49
Marital status			0/13
Married	18 (60%)	27 (77/1%)	
Single	12 (40%)	8 (22/9%)	
Number of pregnancies in married people			
0	9 (50%)	13 (48/14%)	0/26
1	3 (16/6%)	6 (22/22%)	
2	5 (27/7%)	6 (22/22%)	
3	1 (5/5%)	2 (7/4%)	

^*^P < 0.05 was considered statistically significant

^**^significant

The results of this study showed that among the physical symptoms of the PMS in the intervention group compared to the control group, at the end of the study, headache (*p* = 0.001), nausea, constipation diarrhea (*p* = 0.01), swollen (*p* = 0/001) had a significant decrease (Table 3).

**Table 3:** The mean of indicators of physical symptoms of premenstrual syndrome in the control and intervention groups before and after the intervention

Group		Control $\overline{X}\pm SD$	Intervention $\overline{X}\pm SD$	*P* value^*^ Independent t-test
Physical symptoms				
Acne	Before	1/9 ± 1/37	1/85 ± 1/26	0/89
	After	1/26 ± 1/08	1/11 ± 0/9	0/09
	Difference	− 0/66 ± 0/92	− 0/82 ± 1/04	0/5
Bloating	Before	1/34 ± 0/66	1/34 ± 0/88	0/5
	After	0/89 ± 0/43	0/53 ± 0/32	0/08
	Difference	-0/26 ± 0/63	−0/54 ± 0/88	0/01^**^
Breast sensitivity	Before	2/56 ± 0/81	2/74 ± 0/65	0/33
	After	1/90 ± 0/95	1/80 ± 0/93	0/7
	Difference	−0/7 ± 0/98	−1/05 ± 0/9	0/13
Dizziness	Before	2/23 ± 1/19	1/44 ± 1/57	0/06
	After	1/7 ± 1/16	1/20 ± 1/25	0/06
	Difference	−0/4 ± 0/77	−0/4 ± 0/73	0/7
Fatigue	Before	1/56 ± 1/4	1/51 ± 1/37	0/88
	After	1/16 ± 1/17	1/14 ± 1/16	0/8
	Difference	−0/3 ± 0/74	−0/6 ± 0/84	0/13
Headache	Before	1/93 ± 1/36	1/48 ± 1/50	0/21
	After	1/6 ± 1/3	0/85 ± 0/74	0/001^**^
	Difference	−0/5 ± 0/82	−0/42 ± 0/69	0/7
Flushing	Before	2/02 ± 1/21	1/77 ± 1/37	0/42
	After	1/83 ± 1/17	1/31 ± 1/20	0/08
	Difference	−0/36 ± 0/71	−0/8 ± 0/99	0/04^**^
Nausea, diarrhea, constipation	Before	1/90 ± 1/39	1/68 ± 1/45	0/54
	After	1/6 ± 1/24	1/01 ± 0/91	0/01^**^
	Difference	−0/43 ± 0/89	−0/45 ± 0/78	0/9
heart beat (Palpitations)^***^	Before	1/8 ± 1/27	1/08 ± 2/34	0/17
	After	1/4 ± 1/1	1/4 ± 0/97	0/09
	Difference	−0/5 ± 1/00	−0/91 ± 0/98	0/12
Swollen in Breast	Before	1/56 ± 1/38	1/42 ± 1/31	0/68
	After	1/36 ± 1/32	0/98 ± 0/97	0/001^**^
	Difference	−0/33 ± 0/71	−1/00 ± 0/71	0/002^**^
Increased appetite	Before	2/00 ± 1/25	1/97 ± 1/36	0/93
	After	1/7 ± 1/05	1/34 ± 0/99	0/1
	Difference	−0/36 ± 0/71	−0/91 ± 0/88	0/008^**^

^*^P < 0.05 was considered statistically significant

^**^significant

^***^Palpitations are the perceived abnormality of the heartbeat characterized by awareness of cardiac muscle contractions in the chest: hard, fast and/or irregular beats

Also, the comparison of the difference between the mean of symptoms at the beginning and the end of the study showed that among the physical symptoms of the PMS, Bloating (is any abnormal gas swollen, or increase in diameter of the abdominal area) (*p* = 0.01), Swollen in Breast (*p* = 0.002), flushing (*p* = 0.04), increase in appetite (*p* = 0.008) were significantly decreased (Table 3).

## Discussion

Today, solving women’s problems is one of the most important health and research priorities. PMS is one of the most common problems of women, which can interfere with everyday life in women. In this study (8 weeks of aerobic exercise), there was a significant decrease in physical symptoms of premenstrual syndrome. The results of this study showed that among the physical symptoms of the PMS in the intervention group compared to the control group, at the end of the study, headache (*p* = 0.001), nausea, constipation diarrhea (*p* = 0.01), swollen (*p* = 0/001) had a significant reduction. Also, comparison of the difference between the mean of symptoms at the beginning and the end of the study showed that among the physical signs of PMS, Bloating (*p* = 0.01), Swollen (*p* = 0.002), flushing (*p* = 0.04), increase in appetite (*p* = 0.008), had a significant decrease. Although other symptoms also decreased, this was not significant.

Also, the results of Vishnupriya study showed that exercise can reduce symptoms of premenstrual syndrome [11], But Aimee and colleagues reported that there is no significant relationship between exercise and premenstrual syndrome [16]. In 2004, Lustik and colleagues conducted a research on stress, quality of life, physical activity, and symptoms of premenstrual syndrome. This study was performed on 114 women aged 18-33 years old and divided into 2 severe and weak groups in terms of symptoms of premenstrual syndrome. Studies have shown that people who exercise at times experience more severe symptoms than those who often exercise [17, 18].

In the study of Maged et al., reported highly significant difference between the study and control groups weak coordination, confusion, headache, tiredness, pains, tenderness of the breast, and cramps [19].

In the study of El-Lithy et al., the study group showed a significant decrease in all post-treatment subscale symptoms, scores and total score. Haemoglobin, haematocrit, red cell count and platelet count were significantly increased, while mean corpuscular volume (MCV), mean corpuscular haemoglobin (MCH), mean corpuscular haemoglobin concentration (MCHC) and white blood cell count showed no significant differences. There was also a significant decrease in prolactin, oestradiol and progesterone levels. In conclusion, aerobic exercise increases haemoglobin, haematocrit, red cell count and platelet count, and decreases levels of prolactin, oestradiol and progesterone, resulting in improvement of fatigue, impaired concentration, confusion and most premenstrual symptoms [15].

In the study of Azhary et al., reported a significant reduction in low back pain (*p* = 0.001), abdominal pain (*p* = 0.001) and Swollen (*p* = 0.05) [19]. Contrary to the results of this study, In the study of Prior, bloating (*p* = 0.025) decreased significantly [20]. In the study of Fatoukian [21], bloating (*p* = 0.05) showed a significant decrease. Karimian et al. Reported that exercise had a significant effect on abdominal pain (*p* = 0.001) and joint pain (*p* = 0.008) [22]. Contrary to the results of this study, Karimian et al. Reported Bloating and sensitivity of breast (*p* ≤ 0.05) had not recovered after exercise [22], also, Azhary et al. Reported that breast tenderness increased after 8 weeks of aerobic exercise [19]. In confirmation of the results of this study, Abedi and Nikbakht (2007), Moqadasi et al. (2009), Dehghan Manshadi et al. (2008), Samadi et al. (2012) indicate positive effects of exercise on reducing the Physical symptoms of PMS [8, 23–26]. While the study of Beackvid et al. (2000) and Gharakhanlu et al. (2000), Sadler (2010) and Silva (2006) reported that exercise has no effect on the physical symptoms of PMS [12, 13, 27, 28].

Considering that in this study, the severity of symptoms decreased at the end of the study, but this was not significant and only some of the physical symptoms decreased significantly. Due to the difference in the results of this study and other studies, this difference can be attributed to the difference in the duration and intensity of the exercises, the pattern of sleep and nutrition of individuals and their living environment. In fact, the occurrence of electrolyte symptoms such as bloating and swollen may be due to an increase in serum aldosterone, an increase in prostaglandin E2, and a deficiency of vitamin B6 and magnesium. Consequently, given the positive effect of aerobic training on the reduction of serum aldosterone, the results are justifiable [20, 29]. Aerobic exercise lowers renin levels, increases estrogen, progesterone, and reduces serum aldosterone levels, resulting in improved symptoms [30]. Aganoffy considers aerobic exercise ineffective in water retention in athletic and non-athlete women [31]. While Stoddard et al. Showed positive effects of aerobic exercise on the clinical symptoms of PMS, such as pain and water retention [3]. It is said that prolactin levels increase in the end of luteal phase is one of the causes of pain and swollen in the breast. Occurrence of physical symptoms such as swollen, weight gain, headache, breast pain may be related to increased activity in the system of aldesterone renin-angiotensin, prolactin, prostaglandin, deficiency of vitamin B6, E2 and magnesium. Prostaglandin E2 is one of the risk factors for developing physical symptoms. The repeated muscle contraction in physical activity helps to reverse vein and increases the movement of prostaglandin and other substances and prevents their accumulation in the pelvis and reduces the back pain and abdominal discomfort [9]. Khademi et al. Also considered 8 weeks of swimming sport as one of the aerobic exercises that can be used to reduce physical symptoms such as headache, swollen of the breast, and low back pain [32]. The beta-androgen level at the end of the luteal phase decreases due to changes in sex hormones, aerobic exercise results in increased levels of beta-endorphin and increases pain tolerance in individuals, thereby improving the body’s symptoms that are due to decreased beta-endorphin [33]. Increasing renin-angiotogenic activity and decreasing estrogen and progesterone levels have been reported as an effective factor in increasing the serum level of aldesterone in the late phases of the luteal phase. Increasing the serum level of aldesterol leads to increased sodium and water reabsorption and thus causes edema and physical symptoms [34].

Since this syndrome can have negative effects on the efficiency and presence of women in their employment centers, the existence of this syndrome also has a damaging effect on the economic aspect. With the emphasis on the results of the research, exercise can be combined with other therapies to reduce the symptoms of this syndrome.

The lack of control of nutrition, sleep, and unequal motivation of subjects during exercise is a limitation of this study. It is recommended that subsequent studies be conducted with a higher sample size and comparing the types of exercise with aerobic exercise.

## Conclusion

Regarding the results of this study, performing 8 weeks of regular aerobic exercise in people with PMS causes a significant decrease in some of the physical symptoms of the syndrome, For this reason, having a regular exercise program is recommended to all women of reproductive age, especially those with PMS.

## Abbreviation

PMS: Pre Menstrual Syndrome

## References

[1] Hasani N, Kazemi M, Karimi Afshar H, Kazemi M, Tavakoli M (2015). Comparison of the effects of relaxation and vitamin B6 on emotional and physical symptoms in premenstrual syndrome. Evid Based Care J.

[2] Mohebbi Dehnavi Z, Jafarnejad F, Mojahedy M, Shakeri M, Sardar M (2016). The relationship between temperament warm and cold with symptoms of premenstrual syndrome. IJOGI..

[3] Mohebbi-Dehnavi Z, Jaafarnejad F, Kamali Z, Saber Mohammad A (2017). The effect of eight weeks aerobic exercise on psychological symptoms of premenstrual syndrome. Nurs Pract Today.

[4] Lotfi Kashani F, Sarafraz K, Pasha Sharifi H. The effect of musculoskeletal training on reducing symptoms of premenstrual syndrome. Appl Psychol. 2007;2(5)63–74.

[5] Biggs W, Demuth RH (2011). Premenstrual syndrome and premenstrual dysphoric disorder. Am Fam Physician.

[6] Rad M, Sabzevary MT, Dehnavi ZM (2018). Factors associated with premenstrual syndrome in female high school students. J Edu Health Promot.

[7] Mohebbi Dehnavi Z, Rastaghi S, Rad M (2017). A survey on the association of premenstrual syndrome with type of temperament in high school students. IJOGI.

[8] Abedy H, Neksereshgt A, Tashakoriyan F. The effects of resistance and endurance exercise on physical and psychobehavioral symptoms of pre-menstruation syndrome. Pars J Med Sci. 2014;12(3)9–14.

[9] Daley A (2009). Exercise and premenstrual symptomatology: a comprehensive review. J Women's Health (Larchmt).

[10] Omidali F. The effect of Pilates exercise and consuming fennel on pre-menstrual syndrome symptoms in non-athletic girls. Complement Med J. 2015;2(15)1203–1213.

[11] Vishnupriya R, Rajarajeswaram P (2012). Effects of aerobic exercise at different intensities in pre menstrual syndrome published online.

[12] Silva C, Gigante D, Carret M, Fassa A (2006). Population study of premenstrual syndrome. Revista De Saude Publica J.

[13] Sadler C, Smith H, Hammond J, Bayly R, Borland S, Panay N (2010). Lifestyle factors, hormonal contraception and premenstrual symptoms: the United Kingdom Southampton Women’s Survey. J Women's Health.

[14] David A, Bella Z, Berenstein E, Lopes A, Vaisberg M (2009). Incidence of premenstrual syndrome in sport practice. Braz J Sports Med.

[15] El-Lithy A, El-Mazny A, Sabbour A, El-Deeb A (2015). Effect of aerobic exercise on premenstrual symptoms, haematological and hormonal parameters in young women. J Obstet Gynaecol.

[16] Aimee R, Alayne G, Sofija E, Serena C, Biki B, Elizabeth R. Recreational physical activity and premenstrual syndrome in young adult women: a cross-sectional study. PLoS One. 2017;12(1)1–13. 10.1371/journal.pone.0169728 PMC5231278 28081191

[17] Amiri Farahani L, Heidari T, Narenji F, Asghari Jafarabadi M, Shirazi V (2012). Relationship between pre menstrual syndrome with body mass index among university students. J HAYAT.

[18] Maged AM, Abbassy AH, Sakr HRS, Elsawah H, Wagih H, Ogila AI, Kotb A (2018). Effect of swimming exercise on premenstrual syndrome. Arch Gynecol Obstet.

[19] Azhary S, Karimi Chatrudi A, Attarzadeh R, Mazloom R (2005). Officacy of group aerobic exercise program on the intensity of premenstrual syndrome. Iran J Obstet Gynecol Infertil.

[20] Prior JC, Vigna YM. Ovulation disturbances and exercise. Clin Obstet Gynecol. 1991;34(1):180–90. 10.1097/00003081-199103000-00020 2025968

[21] Fatoukian Z, Ghaffari F (2006). The effect of aerobic exercise therapy on symptoms of premenstrual syndrome. J Babol Univ Med Sci.

[22] Karimian N, Rezaeian M, Nassaji F, Velaei N, Gachkar L (2006). The effects of physical activity on premenstrual-syndrome. J Zanjan Univ Med Sci.

[23] Nikbakht M, Ebadi G (2007). The comparison of two premenstrual syndrome (PMS) in high school girls of Ahwaz. Res Sport Sci.

[24] Moqadasi A, Dareh Bidi M, Yosefi M, Kargarfard M (2009). A comparison of prevalence of premenstrual syndrome symptoms between athlete and non-athlete female students. J Sport Exerc Physiol.

[25] Dehghan Monshadi F, Emami M, Ghamkhar L (2008). The effect of three months of regular aerobic exercise on symptoms of premenstrual syndrome. Univ Med Sci.

[26] Samadi Z, Taghian F, Valiani M (2013). The effects of 8 weeks of regular aerobic exercise on the symptoms of premenstrual syndrome in non-athlete girls. Iran J Nurs Midwifery Res.

[27] Beackvid Henri Ksson G, Schnell C (2009). Women edurancerunners with menstrual dysfunction have prolonged in terruption of trainingdue to injury, Linden Hirschbery A: GynecolobstetInVest.

[28] Gharakhanlu R, Kordi M (2000). Study the relationship between menstrual periods, exercise intensity and incidence of sports injuries. Olympic J.

[29] Imami M, Dehghan Monshadi F, Ghamkhar L (1995). Effect of 3 months of aerobic exercise Mnzmbr symptoms of premenstrual syndrome. Institute for Humanities and Cultural Studies Humanities comprehensive portal.

[30] Stoddard JL, Dent CW, Shamcs L, Bernstein L (2007). Exercise training effects on premensterual distress and ovarian steroid hormones. Euro J Appl Physiol.

[31] Aganoffy BG (1994). Aerobic exercise, mood states and mensterual cycle symptomus. J Psychosom Res.

[32] Khademi A, Tabatabaeefar L, Akbari E, Alleyassin A, Ziaee V, Asghari-Roodsari A (2008). Comparison of prevalence of Premenstrualsyndrome in swimmer and non- swimmer students. Acta Medica Iranica.

[33] Charkoudian N, Joyner M (2004). Physiologic considerations for exercise performance in women. Clin Chest Med.

[34] Johnson S (2004). Premenstrual syndrome, premenstrual dysphoric disorder, and beyond: a clinical primer for practitioners. Obstet Gynecol.

